# Camptocormia in an Adolescent: A Case Report and Review of the Literature

**DOI:** 10.1155/2018/4606171

**Published:** 2018-06-05

**Authors:** Laura Kaplan, Erik Aurigemma, Timothy Sullivan, Richard Sidlow

**Affiliations:** ^1^Division of Pediatric Hospitalist Medicine, Department of Pediatrics, Staten Island University Hospital-Northwell Health, Staten Island, NY, USA; ^2^Department of Psychiatry, Staten Island University Hospital-Northwell Health, Staten Island, NY, USA

## Abstract

Camptocormia, or bent-spine syndrome, is an entity with a long history and many etiologies. We discuss below both the history of this diagnosis in light of a rare case of psychogenic camptocormia and the recent changes in nosology regarding this disorder.

## 1. Introduction

Camptocormia, or bent-spine syndrome, is characterized by severe flexion of the thoracolumbar region while standing. Unlike kyphosis, it is neither a fixed nor rigid deformity. The origin of camptocormia is neuromuscular; the bony spine is flexible and the deformity often disappears in the supine position. Ordinary kyphosis is characterized by rigid bony or ligamentous abnormalities of the spine (i.e., osteoporosis, trauma, or spondylitis). In camptocormia, the spine itself is unremarkable.

Today camptocormia is most often ascribed to neuromuscular diseases (Parkinsonism, ALS, myasthenia gravis, and a variety of myopathies) which occur largely in the elderly population. One hundred years ago, the disorder chiefly affected battlefield soldiers and was thought to be a psychological illness, a manifestation of what today might be called posttraumatic stress disorder. Camptocormia is best thought of a symptom complex which may have many etiologies. Psychogenic camptocormia is considered rare today with few cases reported in the literature over the past decade.

## 2. Case Report

A 15-year-old female with a past medical history of obesity presented to the emergency department with a marked 90-degree flexion at the thoracolumbar region. She was able to walk only by using a walker in an unusual manner; she placed her axillae on the hand grips. The patient described constant back pain almost daily for which she took ibuprofen with moderate relief. These symptoms began three years ago following a sexual assault and have progressed significantly. As per the patient's mother, the only time the patient's spine straightened out was during deep sleep. She had no neurologic complaints, specifically denying any sensory or motor deficits, fatigue, ptosis, or any complaints consistent with any form of endocrinopathy. Her family history included siblings with epilepsy, ADHD, and mood disorders.

The patient denied any substance abuse, including use of alcohol or cigarettes, as well as being sexually active. She reported no history of mood or psychotic symptoms and no suicide attempts.

Despite the pain, however, the patient was not concerned with her condition. The patient herself was not interested in physical therapy or additional diagnostic testing or counseling despite her mother's encouragement. She refused a gynecological examination due to anxiety following having been a victim of sexual assault; it was unclear the extent of counseling, if any, she received following the assault.

The patient was a seventh grader at a local school where she participated in extracurricular activities. She reported being well accepted by her peers. She stated having a good social support network including friends and family. Her parents were separated and she lived with her mother and brother.

Physical exam revealed an obese (BMI 39.2) Caucasian female, leaning forward with the spine nearly ninety degrees at the waist. Extremities showed normal strength, sensation, and range of motion. The patient was unwilling to attempt spinal extension. Tenderness to palpation of the paraspinal muscles was present. The rest of the physical examination was unremarkable.

Upon Pediatric Neurology consultation, no evidence of neuromuscular disease was found. Plain radiography of the cervical, thoracic, and lumbar spine was limited due to her deformity, but no abnormalities were noted. Magnetic resonance imaging of the same was unrevealing. Psychiatric consultation was obtained; they concluded that the patient's symptoms were likely manifestations of Conversion Disorder, following exclusion of organic causes.

While she is currently receiving social services support and physical therapy; she declines psychiatric outpatient care at this time.

## 3. Discussion

The first description of camptocormia in western medical literature is often attributed to Brodie in the 1800s. Brodie recognized that spinal pain and curvature could either be due to vertebral trauma or destruction or neuromuscular causes (hysterical spinal neuralgia) [1].

The etiology of back pain and deformity, then as now, may be difficult to determine in a given patient. Although anatomic or structural abnormalities may be found, there is often an emotional component and, occasionally, a component of malingering. This is hardly a new phenomenon. In the mid-19th century, this was common in passengers who suffered back pain from railroad accidents. Treatises from that era discussed the medical, psychiatric, and legal underpinnings of these injuries. Sir William Gowers in 1904 suggested that back pain might be due to fibromuscular inflammation, presaging some of the current thinking about camptocormia [2].

During WWI reports of camptocormia occurring in soldiers were published by Souques [3]. He described soldiers with persistent spinal deformity who had not had direct spinal injuries. Camptocormia was considered a reaction to battlefield stress, “shell shock”, a disorder which included other forms of psychogenic paralysis, tremor, mutism, and fugue states. Shell shock and various conversion symptoms were frequent; some estimates indicated that twenty percent of battlefield soldiers were affected. It was concluded that symptoms were in the patient's mind but often there was little distinction between conversion reactions (unconscious psychological causation of which the patient had neither awareness nor voluntary control) and malingering (where the patient was consciously trying to avoid the return to the battlefield). Souques treated these patients with persuasive electroshock therapy or “torpillage.” This was not the ECT of today; it consisted of administering painful faradic and galvanic shocks repeatedly until the soldiers gave up their symptoms and returned to the battlefield. Some regarded this as simple torture. In the relative peace following WWI, camptocormia was rarely seen but reemerged during WWII [4].

In the twenty-five years following World War II, the condition was rarely discussed in the medical literature. Beginning in the 1960s, scattered reports appeared, initially describing camptocormia as a psychogenic illness. In 1965 an influential paper by Eric Slater disparaged the concept of psychosomatic illness. It was his view that many patients had underlying organic disorders which were not yet diagnosed (“the diagnosis of ‘hysteria' is a disguise for ignorance and a fertile source of clinical error”) [5].

In 1987 the first description of camptocormia secondary to an organic cause was published, a case report of transient camptocormia secondary to a valproate overdose [6]. Subsequently, Laroche demonstrated paraspinal myositis on spinal CT scans of patients with camptocormia. Other investigators detected abnormalities on muscle biopsy and EMGs. By 1995, Laroche et al. proposed that camptocormia, rather than a psychogenic illness, was often a myopathy affecting the spinal extensors [7]. In 1999, the association of Parkinson's disease with camptocormia was described by Djaldetti et al. [8]. Subsequent reports have strengthened the relationship of Parkinsonism to camptocormia as well implicating a variety of other neuromuscular causes including myotonic dystrophy, dystonia, myopathies, myasthenia gravis, ALS, and drugs. Magraf et al. [9] have recently provided a helpful review of the literature, focusing on neuromuscular pathologies and syndromes [9].

Since 2005, only two cases of psychological camptocormia have been reported in PubMed. Bayazit et al. and Skidmore et al. described it in patients with schizophrenia and bipolar disease, respectively. [10, 11]. It is important to note, however, that the diagnosis in these cases may be confounded as both patients were also on classes of psychoactive drugs known to cause camptocormia.

Psychogenic camptocormia was most often described in male soldiers involved in stressful conflict. Depression has also been suggested as a possible etiologic factor. [12]. Massa described camptocormia in a patient with underlying depression whose symptoms resolved with psychotherapy and amitriptyline. The first reports of women with the disease were not until 1961. Domestic abuse may have contributed to the disorder in some patients. Children and adolescents have rarely been affected. In 2000 Pfeiffer and von Moers reported the first case in an individual under 17 years of age, occurring in a 13-year-old boy. The authors felt this was due to his traumatic immigration away from his birthplace and hinted at severe parental violence and conflict [13]. Several years later, Rajmohan 2004 described the ailment in a 15-year-old girl with oppositional defiant disorder and borderline mental retardation. They noted the patient was receiving secondary benefits, that is, constant reassuring parental attention and care [14].

Since the late 19th century, a group of movement and gait disturbances has been described for which there are no known structural, neurologic, or muscular abnormalities. As in camptocormia, these disorders were assumed to have a psychiatric basis and termed “psychogenic movement disorders.” Commonly referred to as Conversion Disorders, they are classified under “Somatic Symptom and Related Disorders,” in DSM-5. While a majority of patients may have psychological factors, not all patients have a demonstrable psychiatric illness. Functional movement disorder (FMD) is currently the preferred term for this group of conditions, recognizing that the pathophysiology of these disorders is not fully known. FMD presents with a variety of syndromes, functional tremor, dystonia, gait disturbance, myoclonus, startle reactions and functional Parkinsonism. Astasia-abasia (Blocq's disease), and the psychogenic inability to stand upright or walk, and was one of the first functional gait disorders described [15, 16].

Psychogenic camptocormia may also be regarded as a member of the Functional Movement Disorder group or a Conversion Disorder. Utilizing this concept, while our patient's particular manifestation of “camptocormia'” is unusual, FMD and conversion reactions are far more common. The American Psychiatric Association estimates that “Somatic Symptom Disorder” affects 5-7% of the population, including children. Females are more often affected than males. Physical and sexual violence may be an important factor in women who manifest somatic symptoms and illness [17].

The manifestations of Conversion Disorders may be influenced by cultural norms or social expectations. Brown and Lewis-Fernández note that the symptoms expressed may be influenced by culture. Manifestations of culturally shaped Conversion Disorders include increased frequency of pseudoneurologic syndrome in the Turkish population; paralysis in the Dutch; and sensations of being infested by ants or worms in Nigerians [18].

Additionally, there are a number of very specific “culture-bound syndromes”, which mimic some of the behaviors of conversion reactions: “speaking in tongues and manifestations” (Pentecostal Church adherents); “falling-out” (Caribbean community),* ataque de nervios* (certain Hispanic populations), and* kyol goeu* (Cambodian). The societies in which these syndromes appear to affect members often accept these behaviors as normal reactions to stress [18]. In that regard, shell shock (including camptocormia) was so prevalent in WWI that it approached a cultural norm.

The availability of fMRI and its increasing use in the study of a variety of poorly understood psychiatric illnesses and syndromes has resulted in intriguing insights into the neurophysiologic nature of conversion symptomatology [Mehta et al. 2013]. In a recent study of 23 patients with functional neurologic disorder (FND), childhood abuse and PTSD, for example, Perez et al. described significant volumetric abnormalities in the anterior cingulate cortex (ACC) and insula, regions that comprise the salience network and are thought to be involved in the pathophysiology of these disorders [19].

In a review of the literature on the neurophysiology and neuroanatomy of FND, Voon et al. concluded that that FND results “from a mix of higher-order influences (e.g., attention to self or expectation) and bottom-up limbic influences (e.g., trauma and arousal) interacting with and influencing basic motor function (e.g., intention, inhibition), implicating complex associative regions and processing upstream of primary motor and sensory cortices” [20]. Research into these complex, costly, and disabling disorders may not only lead to better treatments, but importantly, also to their destigmatization.

## 4. Conclusion

While organic neuromuscular disorders are usually the cause of camptocormia in most patients, the etiology may also, if rarely, be psychiatric. Patients presenting with this finding require a full history and neurologic workup to exclude neuromuscular disease, often including CNS imaging, EMG, and muscle biopsy. When no abnormalities are identified, a form of Somatic Symptom Disorder must be considered. Conversion Disorders commonly appear in the aftermath of stressful life events, especially sexual trauma, which in rare instances may manifest as camptocormia.
